# Application value of elbow arthrography in the treatment of chronic radial head dislocation in children

**DOI:** 10.3389/fped.2025.1645613

**Published:** 2025-11-14

**Authors:** Hai Jiang, Tao Li

**Affiliations:** Pediatric Orthopedics Department, Northwest Women’s and Children’s Hospital, Xian, China

**Keywords:** elbow arthrography, children, radial head, dislocation, diagnosis

## Abstract

**Objective:**

To investigate and analyze the application value of elbow arthrography in the treatment of chronic radial head dislocation in children.

**Methods:**

From January 2014 to January 2017, 15 children with chronic anterior radial head dislocation were treated (11 boys, 4 girls; average age 9 years, range 3–11 years). Intraoperative elbow arthrography was performed to assess the position of the radial head relative to the joint capsule. The nature of the dislocation was confirmed by opening the joint capsule to directly observe the relationship between the radial head and capitellum, as well as the morphology of the radial head. Reduction of the radial head was achieved via proximal ulnar osteotomy, and fixation was performed using a plate and screws with or without Kirschner wires (K-wires).

**Results:**

Elbow arthrography showed the radial head was located within the joint capsule in 12 cases and outside the capsule in 3 cases. Among the cases: All 8 congenital dislocations had the radial head within the joint capsule. Of the 7 traumatic dislocations, 4 had the radial head within the capsule and 3 outside. Direct inspection after capsulotomy confirmed the arthrography findings and revealed characteristic morphological differences. In congenital dislocations, there was no scar tissue between the radial head and capitellum, and the radial head fovea was shallow and flat. In traumatic dislocations, obvious scar tissue was present between the radial head and capitellum, and the radial head fovea was distinct. Three patients with traumatic dislocations (all with the radial head outside the capsule on arthrogram) required supplemental trans-articular K-wire fixation due to instability after reduction. Follow-up ranged from 9 months to 4 years (average 2 years 5 months). Postoperative radiographs showed no cases of radial head re-dislocation or subluxation. Postoperative elbow range of motion improved in all patients, with no reports of pain or functional instability.

**Conclusion:**

Elbow arthrography serves as a valuable adjunct for differentiating between congenital and traumatic radial head dislocations, thereby informing both surgical strategy and the decision for supplemental fixation. When stable bony alignment is achieved, proximal ulnar osteotomy without annular ligament reconstruction represents an effective management strategy for chronic radial head dislocation in children.

## Introduction

Chronic radial head dislocation in children arises either as a sequela of missed Monteggia fracture (1–3) or as a congenital condition (4). This pathology can lead to progressive valgus deformity of the affected elbow and impair its function. Over time, longitudinal growth discrepancy may develop, resulting in relative overgrowth of the radius compared to the ulna. Consequently, the ulna on the affected side becomes shorter than that on the contralateral, unaffected side (5). Treatment of chronic radial head dislocation is challenging (6–9). Among the clinical approaches available, proximal ulnar osteotomy, with or without gradual lengthening, is the most frequently recommended procedure. However, numerous postoperative complications can occur throughout the treatment course (10). Achieving satisfactory outcomes remains a significant challenge for pediatric surgeons.

Due to the unclear history in children, distinguishing between traumatic and congenital etiologies can be challenging. This study utilized intraoperative elbow arthrography to indirectly assess the relationship between the radial head and the joint capsule as an indicator of etiology (traumatic vs. congenital), which was subsequently verified by direct observation upon opening the capsule. Treatment involved proximal ulnar osteotomy for radial head reduction, followed by plate and screw fixation with or without Kirschner wires (K-wires). Satisfactory short-term outcomes were achieved, as reported below.

## Materials and methods

Fifteen children with anterior radial head dislocation were treated between January 2014 and January 2017 (11 boys, 4 girls; average age 9 years, range 3–11 years). Dislocation involved the left side in 3 cases and the right side in 12 cases. All dislocations were anterior.

Preoperative imaging included anteroposterior (AP) and lateral views of the affected elbow. Intraoperatively, the child was placed supine. After anesthesia induction and standard sterile preparation and draping, posterior or lateral elbow arthrography was performed. A 5 ml syringe needle was used for puncture. After confirming intra-articular placement, approximately 1 ml of iohexol (Omnipaque) contrast agent was injected. Fluoroscopy was used to visualize the position of the radial head. A posterior elbow approach was used: an incision was made along the lateral border of the ulna, dissecting through skin and subcutaneous tissue to reach the proximal ulna. The incision was extended laterally, the joint capsule was opened, and the radial head was exposed. An oscillating saw was used to perform an osteotomy from posterior to anterior at the proximal ulna, near the level of the coronoid process. The proximal ulnar segment was flipped. Any scar tissue between the radial head and capitellum was thoroughly debrided. The proximal ulna was then rotated or angulated posteriorly to reduce the radial head. With the radial head satisfactorily reduced, the osteotomy site was fixed using a plate and screws with or without K-wires. Forearm pronation and supination were tested to assess radial head stability. For cases unstable in pronation, a percutaneous Kirschner wire was inserted through the capitellum to fix the radial head with the elbow flexed at 90°, in a supinated position. No annular ligament reconstructions were performed in this series.

## Results

Preoperative radiographic assessment revealed differences between the two etiologies, summarized in Table 1. Congenital dislocations typically presented with an increased carrying angle, an irregular and flattened radial head, overlap of the radial head and distal humerus on AP view, and a flattened capitellum. Traumatic dislocations generally had a normal carrying angle, a relatively normal radial head shape, and a prominent capitellum.

**Table 1:** Preoperative radiographic features.

Feature	Congenital dislocation (*n* = 8)	Traumatic dislocation (*n* = 7)
Carrying angle	Significantly increased	No significant change
Radial head morphology	Irregular, flattened	Normal
Radial head position (AP view)	Overlaps distal humerus	Variable
Capitellum morphology	Flattened	Prominent
Proximal ulna posterior border	Flattened	Angulated anteriorly

Elbow arthrography showed the radial head within the joint capsule in 12 cases and outside the capsule in 3 cases. Regarding capsule position: All 8 congenital cases had the radial head within the capsule. Among the 7 traumatic cases, 4 had the radial head within the capsule and 3 outside. Direct inspection after capsulotomy confirmed these relationships and revealed characteristic findings: In the 8 congenital dislocation cases, there was no scar tissue between the radial head and capitellum, and the radial head fovea was shallow and flat. In the 7 traumatic dislocation cases, significant scar tissue was present between the radial head and capitellum, and the radial head fovea was distinct.

Arthrography was helpful for the surgical decision. When the dislocation was confirmed congenital, the proximal ulnar rotation osteotomy was performed. When the dislocation was detected traumatic, the posterior bending correction osteotomy of proximal ulna was performed. However, in 3 of the 7 traumatic cases (all of which had the radial head outside the capsule on arthrogram), intraoperative testing revealed persistent instability after reduction, necessitating supplemental trans-articular K-wire fixation. Supplemental trans-articular K-wire fixation was used in 2 of the 8 congenital cases because of instability during forearm pronation.

The K-wire stabilizing the radial head was typically removed 4 weeks postoperatively. Removal of the plate and screws was determined based on radiographic evidence of osteotomy union. All osteotomies healed primarily. Follow-up ranged from 9 months to 4 years (average 2 years 5 months). Follow-up radiographs showed no instances of radial head re-dislocation or subluxation. No intraoperative or postoperative complications, such as nerve injury, infection, or nonunion, were observed. While formal functional scores were not used, clinical assessment at final follow-up indicated improved elbow range of motion in all patients, with no reports of pain or functional instability.

**Figure 1:**
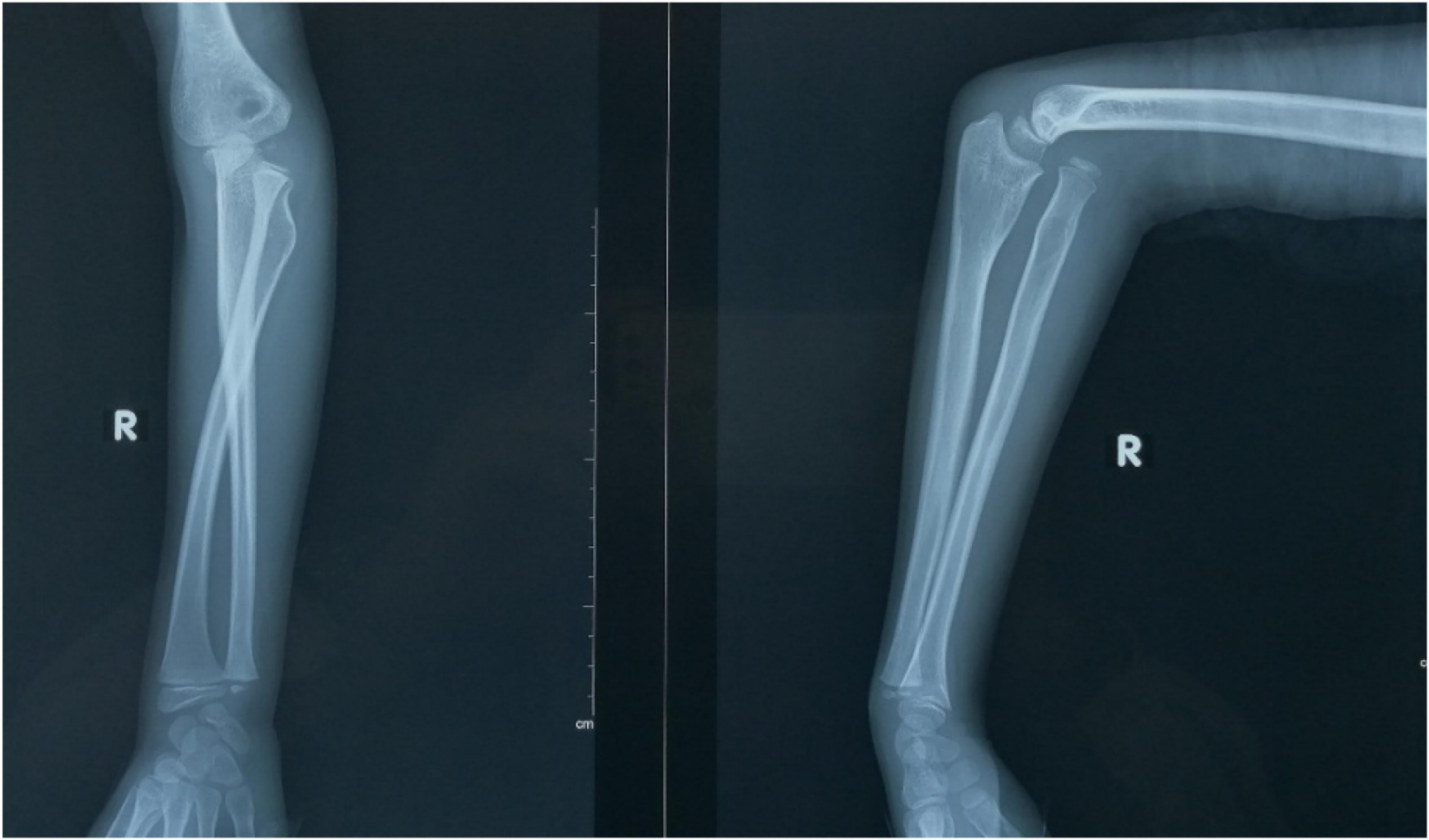
This was an anterior dislocation of the radial head. There was a “ulnar bow sign” in the affected ulna.

**Figure 2:**
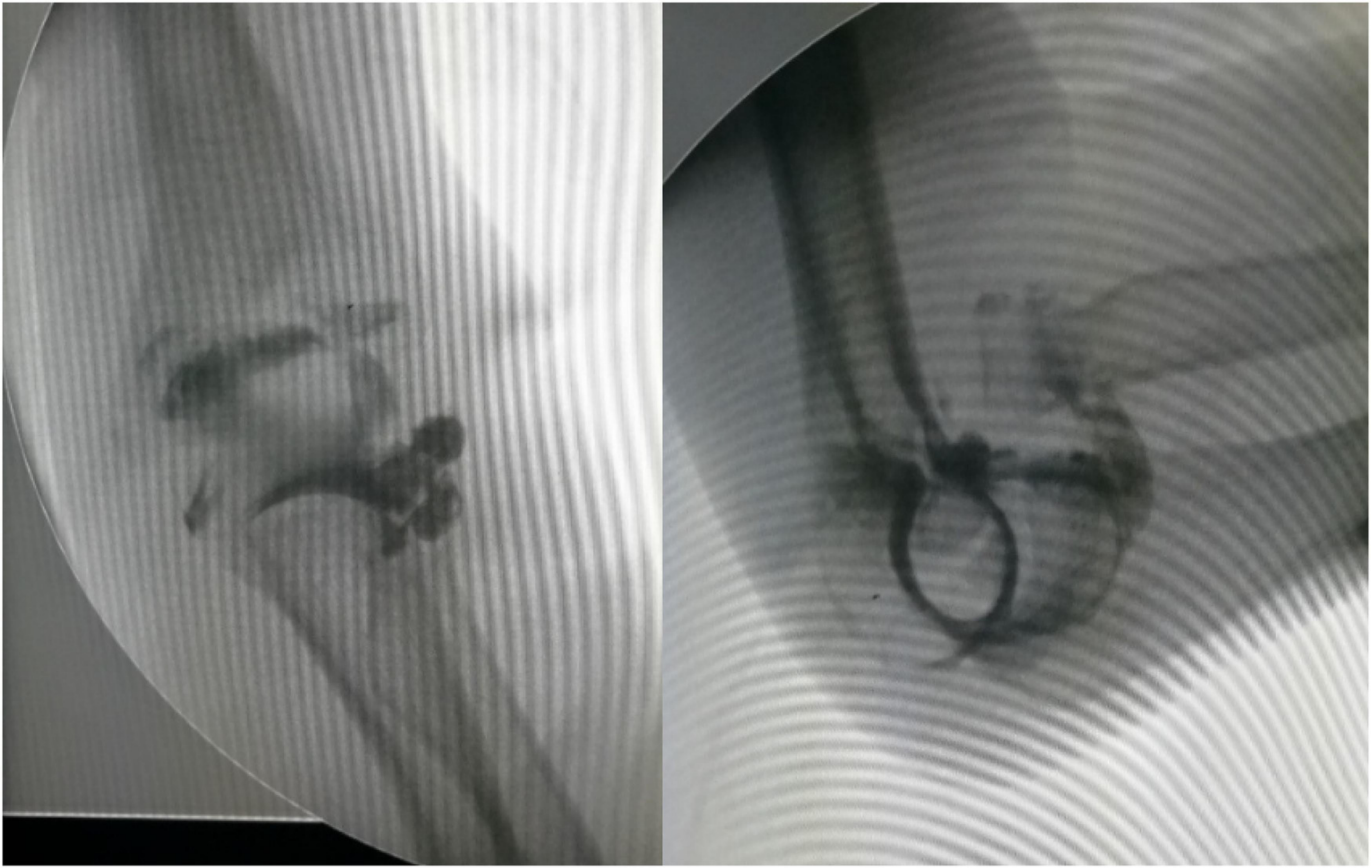
Intraoperative elbow arthrography confirmed the radial head remained within the joint capsule.

**Figure 3:**
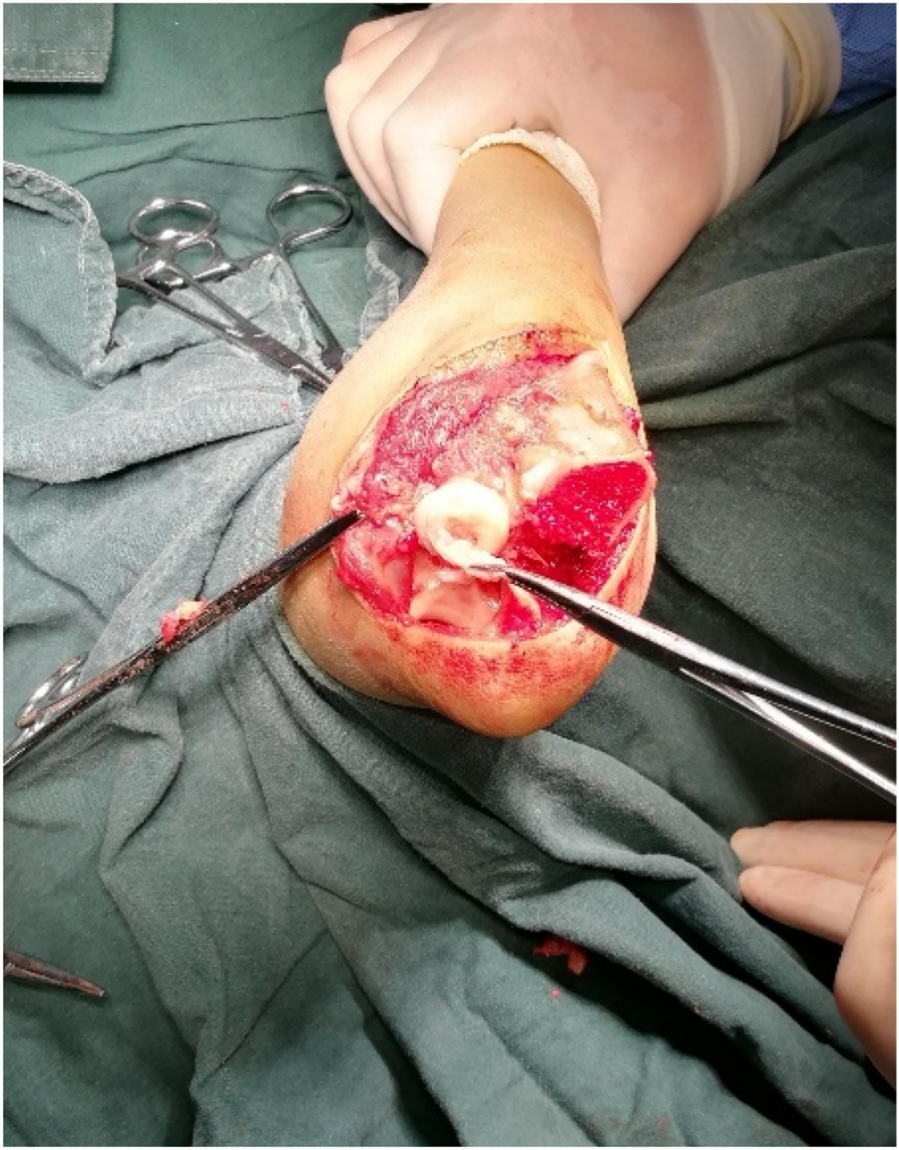
Upon capsulotomy, scar tissue was observed around the radial head, though the depression of the radial head maintained a normal morphology.

There were two typical patients. An 11-year-old boy presented with a 5-year interval between elbow trauma and surgical intervention. Intraoperative elbow arthrography confirmed the radial head remained within the joint capsule. Upon capsulotomy, scar tissue was observed around the radial head, though the depression of the radial head maintained a normal morphology (Figures 1–3).

**Figure 4:**
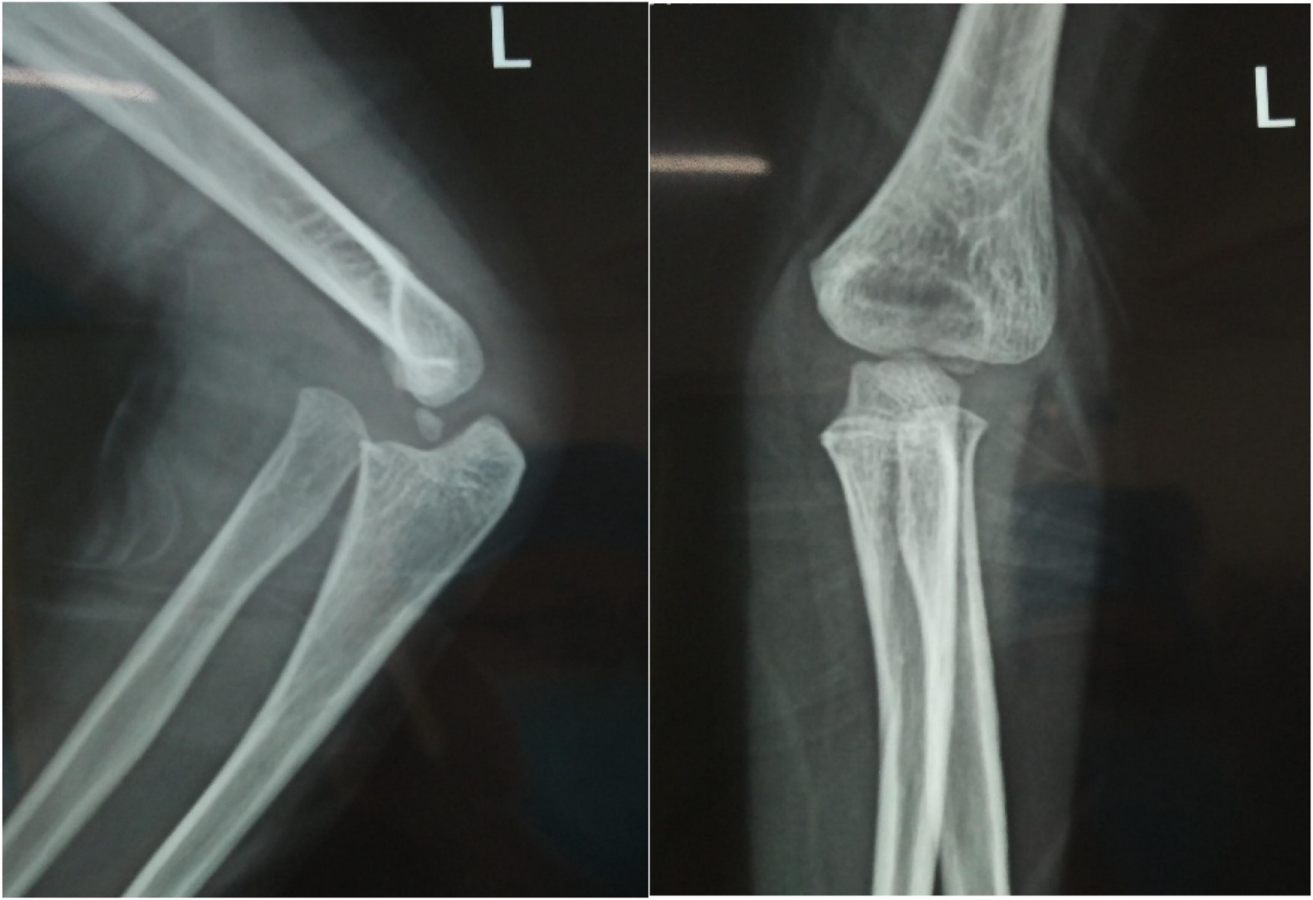
The x-ray demonstrated the anterior dislocation of the radial head.

**Figure 5:**
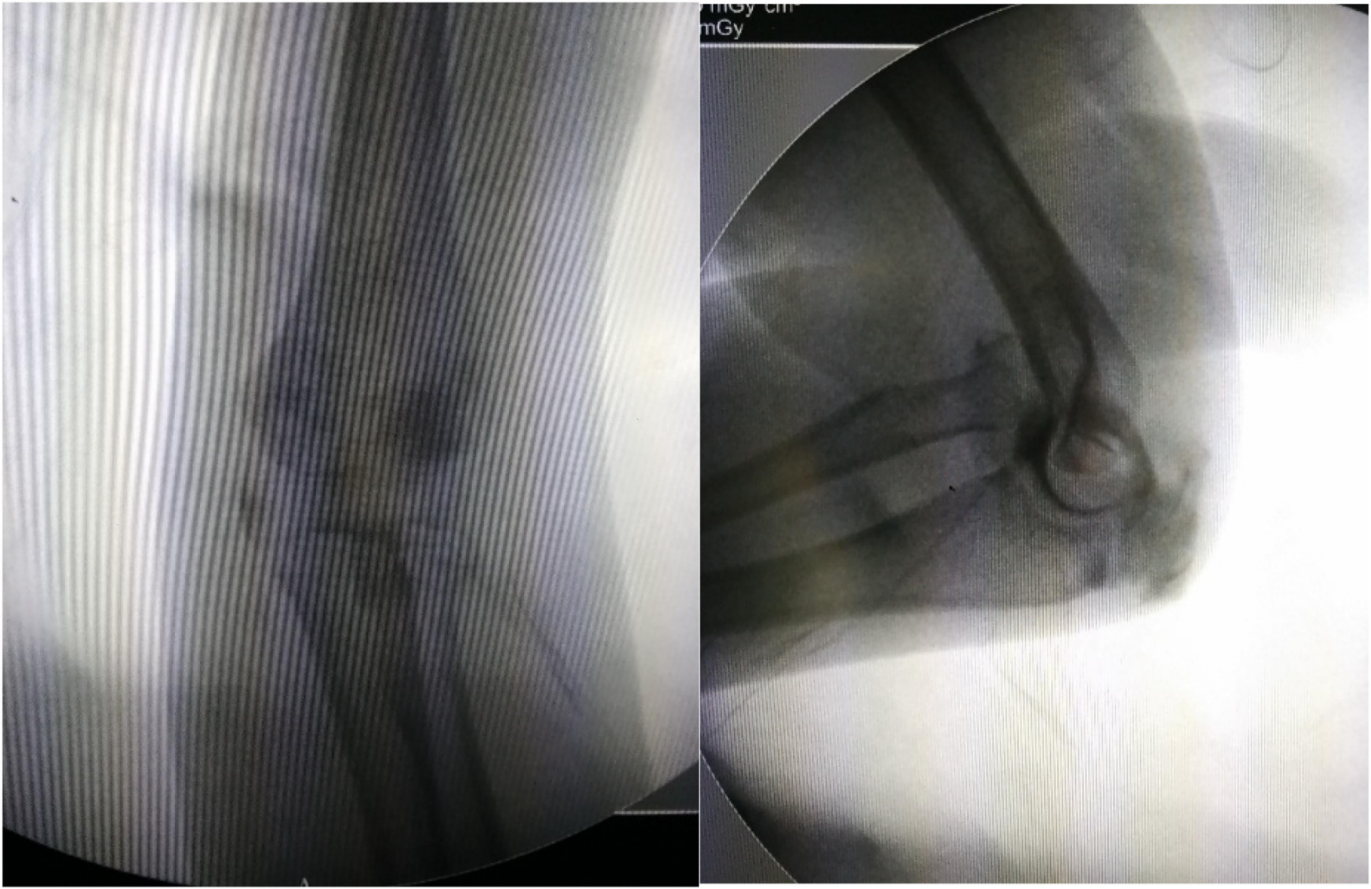
Intraoperative arthrography also showed the radial head within the capsule.

**Figure 6:**
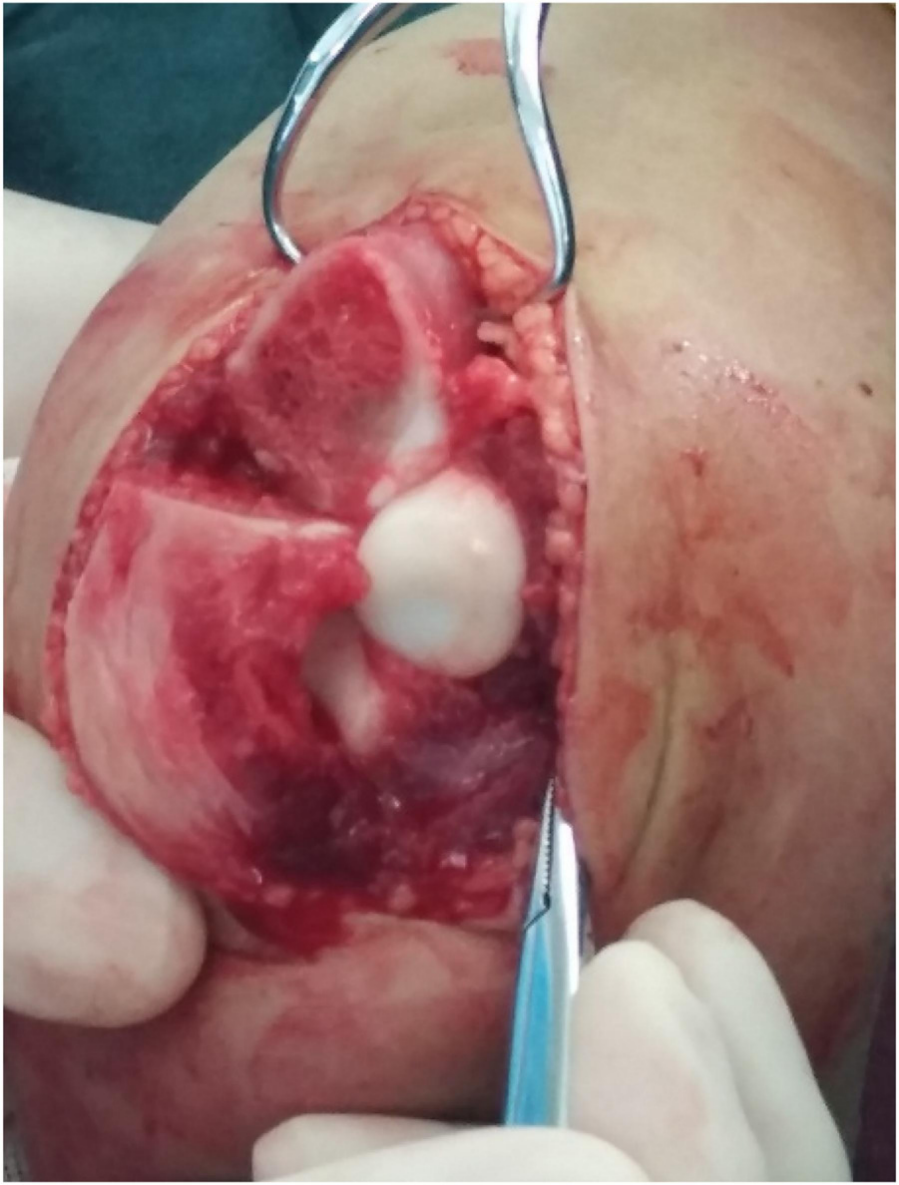
Capsulotomy revealed no scar tissue between the radial and humeral heads, but the radial head depression was shallow and flattened.

**Figure 7:**
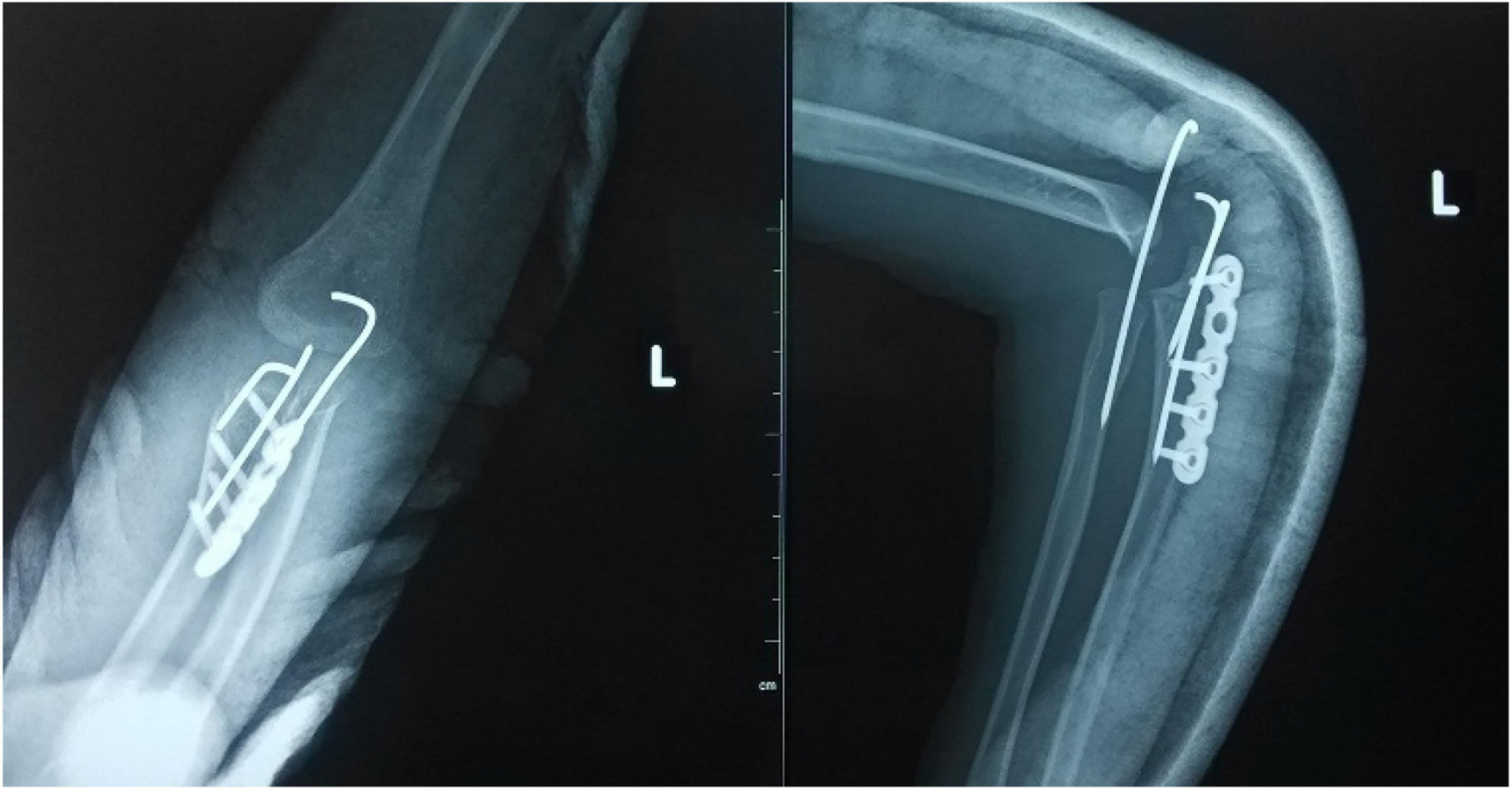
Proximal ulnar osteotomy was performed with plate and K-wires internal fixation. Supplemental trans-articular K-wire fixation was performed because of the instability in the forearm pronation movement.

**Figure 8:**
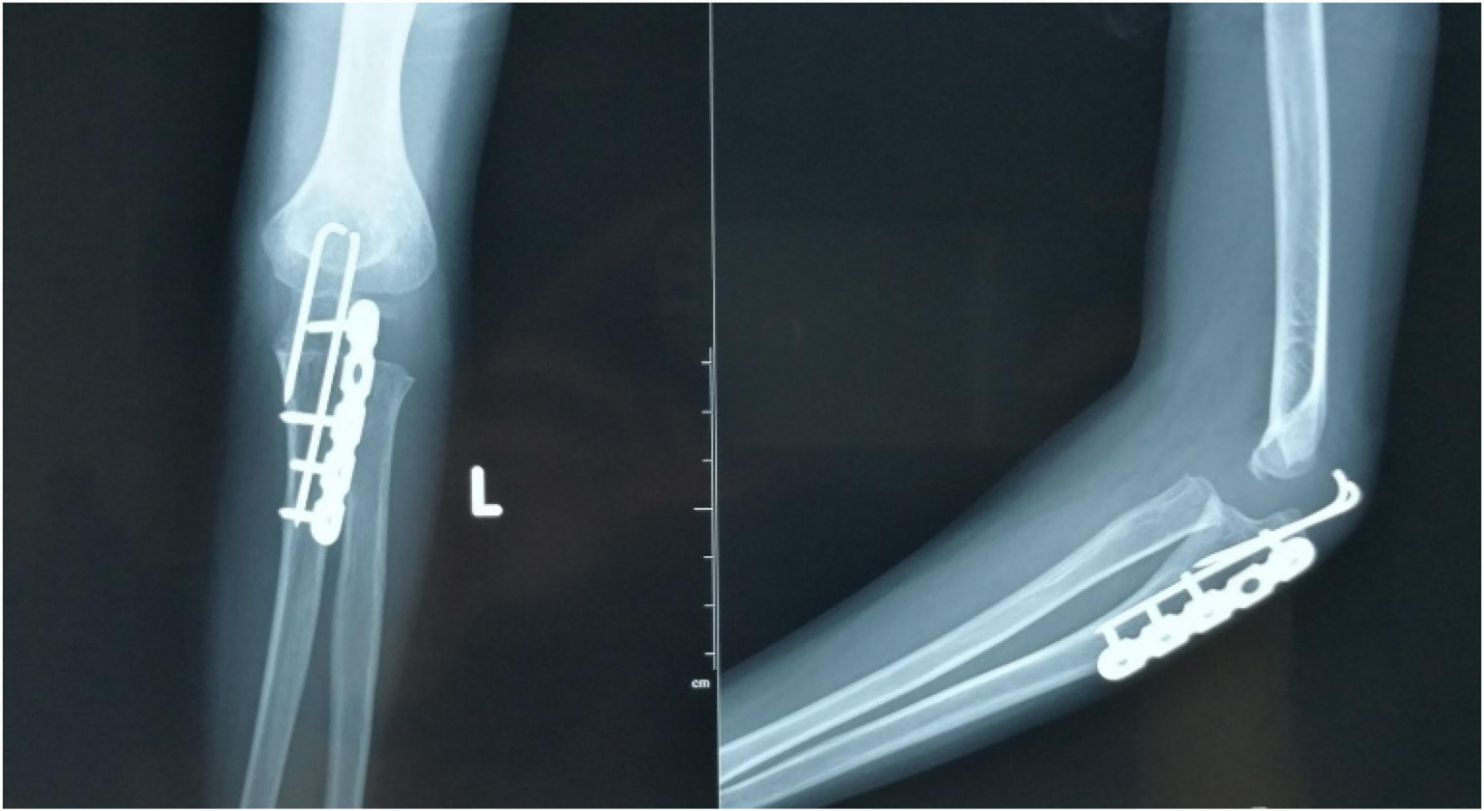
The bony union was achieved at 6 months postoperatively.

**Figure 9:**
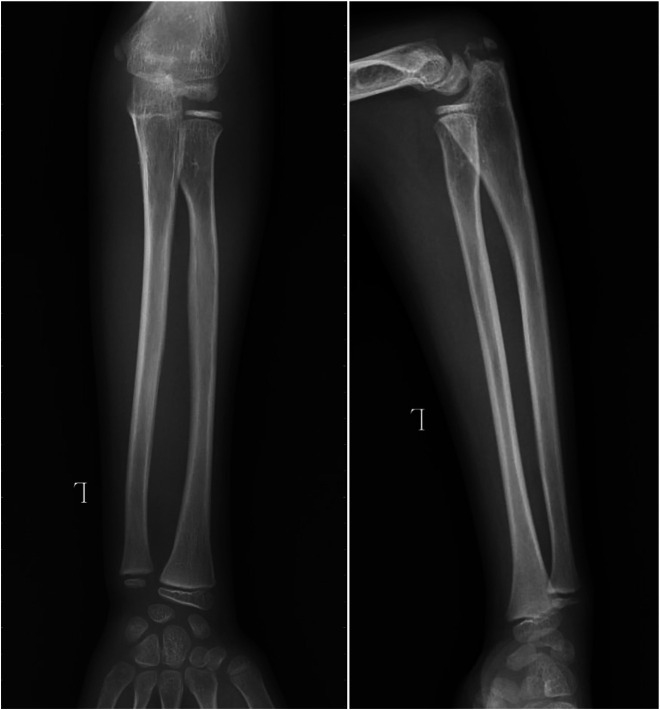
Four-year follow-up demonstrated optimal realignment of the radial head within the elbow joint.

A 5-year-old boy had no history of left elbow trauma. Intraoperative arthrography also showed the radial head within the capsule. Capsulotomy revealed no scar tissue between the radial and humeral heads, but the radial head depression was shallow and flattened (Figures 4–6). Proximal ulnar osteotomy was performed with plate and K-wires internal fixation (Figure 7), with bony union achieved at 6 months postoperatively (Figure 8). Four-year follow-up demonstrated optimal realignment of the radial head within the elbow joint (Figure 9).

## Discussion

Monteggia fracture in children involves a fracture of the ulna combined with radial head dislocation. It is frequently missed initially, leading to chronic radial head dislocation. Anterior dislocation is more common clinically than lateral or posterior dislocation. The dislocated radial head, lacking the constraints of the capitellum and proximal ulna, develops abnormally, often becoming deformed. Long-standing post-traumatic radial head dislocation can present similarly to congenital dislocation. Without a clear history, differentiation is difficult.

In our series, 4 of the 7 traumatic cases had a definite history of trauma, while the other 3 had unclear histories. All 7 traumatic dislocations had been present for over 1 month (longest duration 5 years). None of the 8 congenital cases had a definite trauma history. Specific post-injury symptoms could not be reliably assessed due to the chronic nature in all cases. No associated deformities were found in any of the 15 patients. Preoperative closed reduction attempts were unsuccessful in all 15 cases.

Elbow arthrography is commonly used to diagnose elbow fractures in children with unossified epiphyses, aiding differentiation between distal humeral epiphyseal separation, lateral condylar fractures, and elbow dislocations (11–14). Its use specifically for assessing radial head dislocation in children is less frequently reported domestically or internationally (15, 16). In this study, intraoperative arthrography revealed that in congenital dislocations, the radial head and capitellum resided within the same joint capsule, surrounded by contrast. Traumatic dislocations presented two scenarios. In acute dislocations with significant ulnar angulation, arthrography showed the radial head outside the joint capsule relative to the capitellum, without surrounding contrast. In contrast, in gradual dislocations due to minimal ulnar angulation or greenstick fracture, arthrography showed the radial head within the joint capsule. Therefore, elbow arthrography provides auxiliary value in differentiating the nature of radial head dislocation. The findings of this study indicate that intraoperative elbow arthrography constitutes a valuable adjunct in the surgical management of pediatric chronic radial head dislocation. Its principal utility lies in enabling real-time, indirect assessment of the spatial relationship between the radial head and the joint capsule, thereby providing insight into the etiology. This imaging modality was especially diagnostic in scenarios where the patient history was ambiguous. Meanwhile, as noted in the results, arthrography guided the choice of osteotomy technique.

The traditional treatment for congenital radial head dislocation in children is radial head excision in adulthood, which may alleviate pain but does not significantly improve elbow flexion/extension or forearm rotation (17). Recent literature increasingly advocates for early surgical intervention in children (18, 19). Early reduction allows the radial head to remodel and develop (20), improving congruence with the capitellum and proximal ulna, potentially leading to a more functional elbow joint. Outcomes are generally superior to delayed excision. Techniques reported include proximal ulnar osteotomy, with or without annular ligament reconstruction, and radial shortening (20, 21). We achieved satisfactory results using isolated proximal ulnar osteotomy for reduction, without annular ligament reconstruction. We believe that precise correction of the bony deformity is paramount for maintaining radial head reduction.

We intentionally did not perform annular ligament reconstruction as part of our surgical approach. Our core principle was that a stable, anatomical reduction of the radial head via precise ulnar osteotomy was the foundation of treatment. In our cohort, this method-using temporary K-wire fixation only if instability was detected after the bony correction-produced excellent radiographic outcomes with no re-dislocations. This success indicates that ligament reconstruction is likely unnecessary when stable bony alignment is achieved, which simplifies the surgery and can reduce morbidity. Our results align with a growing body of literature that supports the efficacy of isolated bony procedures (18, 20).

Operative findings corroborated the value of arthrography by revealing a clear pathological profile. Congenital cases were uniformly characterized by a lack of scar tissue and a shallow, flat radial head fovea. In contrast, traumatic cases invariably featured scar tissue and a distinct fovea. This morphological distinction, supported by preoperative radiographic differences (Table 1), was dynamically confirmed by arthrography during surgery. Furthermore, we noted that an extracapsular position of the radial head, identified intraoperatively in three traumatic cases, was correlated with more severe instability-all three required trans-articular K-wire fixation. Consequently, the arthrography finding of an extracapsular radial head appears to be a valuable indicator of the potential need for additional stabilization.

This study has several limitations. Its retrospective, single-center design and small sample size limit the generalizability of the findings. The gender distribution is imbalanced. The lack of a control group managed without arthrography prevents a direct measurement of its added value. Furthermore, the assessment of functional outcomes was not standardized with validated scores, relying instead on clinical observation and absence of complications. Future prospective, multi-center studies with larger cohorts, long-term follow-up, and detailed functional assessments are warranted to further validate these findings.

## Conclusion

Elbow arthrography serves as a valuable adjunct for differentiating between congenital and traumatic radial head dislocations, thereby informing both surgical strategy and the decision for supplemental fixation. When stable bony alignment is achieved, proximal ulnar osteotomy without annular ligament reconstruction represents an effective management strategy for chronic radial head dislocation in children.

## Data Availability

The raw data supporting the conclusions of this article will be made available by the authors, without undue reservation.
